# Autoimmunity-Associated SNP rs3024505 Disrupts STAT3 Binding in B Cells, Leading to IL10 Dysregulation

**DOI:** 10.3390/ijms251810196

**Published:** 2024-09-23

**Authors:** Aksinya N. Uvarova, Elina A. Zheremyan, Alina S. Ustiugova, Matvey M. Murashko, Elvina A. Bogomolova, Denis E. Demin, Ekaterina M. Stasevich, Dmitry V. Kuprash, Kirill V. Korneev

**Affiliations:** 1 Center for Precision Genome Editing and Genetic Technologies for Biomedicine, Engelhardt Institute of Molecular Biology, Russian Academy of Sciences, 119991 Moscow, Russia; 2 Laboratory of Intracellular Signaling in Health and Disease, Engelhardt Institute of Molecular Biology, Russian Academy of Sciences, 119991 Moscow, Russia; 3 Moscow Center for Advanced Studies, 123592 Moscow, Russia

**Keywords:** IL10, B cells, STAT3, regulatory SNP, autoimmune diseases, immunoregulation

## Abstract

Interleukin 10 (IL10) is a major anti-inflammatory cytokine that acts as a master regulator of the immune response. A single nucleotide polymorphism rs3024505(C/T), located downstream of the *IL10* gene, is associated with several aggressive inflammatory diseases, including systemic lupus erythematosus, Sjögren’s syndrome, Crohn’s disease, and ulcerative colitis. In such autoimmune pathologies, IL10-producing B cells play a protective role by decreasing the level of inflammation and restoring immune homeostasis. This study demonstrates that rs3024505 is located within an enhancer that augments the activity of the IL10 promoter in a reporter system based on a human B cell line. The common rs3024505(C) variant creates a functional binding site for the transcription factor STAT3, whereas the risk allele rs3024505(T) disrupts STAT3 binding, thereby reducing the IL10 promoter activity. Our findings indicate that B cells from individuals carrying the minor rs3024505(T) allele may produce less IL10 due to the disrupted STAT3 binding site, contributing to the progression of inflammatory pathologies.

## 1. Introduction

Autoimmune diseases characterized by aberrant immune responses against self-antigens pose significant challenges in clinical management due to their chronic nature and complex etiology [1,2,3]. Among the critical regulatory components of the immune system is interleukin 10 (IL10), a cytokine known for its potent anti-inflammatory properties [4,5,6]. Even though different subsets of T cells, monocytes, and macrophages are considered to be the main sources of IL10, regulatory B cells with high potency for IL10 production have also been found to play a pivotal role in the maintenance of immune homeostasis and protection against the detrimental effects of autoimmune responses through their anti-inflammatory actions [7,8,9,10,11]. IL10 and IL10 receptor deficiencies lead to severe intestinal inflammation and early-onset inflammatory bowel disease in both mice and humans, highlighting the importance of IL10 signaling in maintaining intestinal mucosal homeostasis [12,13]. Resident microbiota activates IL10-producing regulatory B cells through TLR2, MyD88, and PI3K pathways, which help maintain colonic homeostasis and reduce T cell activation [14], which is critical in the context of inflammatory bowel disease, including Crohn’s disease and ulcerative colitis. In the pathogenesis of such severe autoimmune diseases as type 1 diabetes (T1D) and systemic lupus erythematosus (SLE), IL10 plays a multifaceted role. Some studies showed that IL10 can be linked to a tolerant state of regulatory IL10-producing B cells in T1D patients; however, it can exhibit a pathogenic action at the onset of the disease [15]. During SLE, IL10-producing B cells contribute to autoantibody production and tissue damage, while other studies indicate that they play a regulatory role in suppressing disease and immune responses [16,17]. SLE B cells display defective TLR9 responses, leading to the diminished production of anti-inflammatory IL10, which may contribute to the breakdown of B cell tolerance and the persistence of autoreactive B cells in SLE [18]. Negative correlations have been observed between IL10-producing regulatory B cell numbers and disease activity in patients with Sjögren’s syndrome [19]. Understanding the regulatory mechanisms governing IL10 expression is essential for elucidating the pathophysiology of autoimmune diseases and identifying potential therapeutic targets. It has been shown that genetic variations (in most cases, single nucleotide polymorphisms, SNP) of non-coding regions play a functional role in gene expression and susceptibility to various diseases [20,21,22]. There is a list of polymorphisms located in the regulatory elements of the *IL10* gene that have been implicated in the regulation of its expression [23,24,25]. The single nucleotide polymorphism rs3024505 is located in an intergenic region proximal to the 3′-untranslated region of the *IL10* gene [26]. The rs3024505(T) allele may be a risk factor in inflammatory bowel diseases (Crohn’s disease and ulcerative colitis) in the Han Chinese, Australian/New Zealand, and European populations [26,27,28,29,30,31]. Moreover, rs3024505(T) has been shown to be associated with SLE among Swedish and American populations [32,33] and with Sjögren’s syndrome among Italian individuals [34] (Table S1). On the other hand, the major rs3024505(C) variant is considered a risk factor for T1D among British populations and North Americans [35,36]. Investigating how genetic variations influence IL10 expression in B cells is vital for understanding their role in disease progression. Our study focuses on elucidating the functional effects of the rs3024505 SNP on IL10 expression in human B cells.

## 2. Results

### 2.1. The Risk T Allele of rs3024505 Abolishes IL10 Enhancer Activity in B Cell Line Reh

The polymorphism rs3024505(C/T), which is an eQTL (expression quantitative trait loci) for the *IL10* gene [37], is located in the potential enhancer [38] (chr1:206766108-206767105; GRCh38/hg38) downstream of this gene (Figure S1). This genomic region has epigenetic marks of enhancer regulatory elements—high levels of H3K4me1 and H3K27ac histone modifications, DNase I hypersensitivity sites, and clusters of transcription factor binding sites [39]. The minor rs3024505(T) allele frequency ranges from 0.026 to 0.164, depending on the population (Table S2).

**Figure 1:**
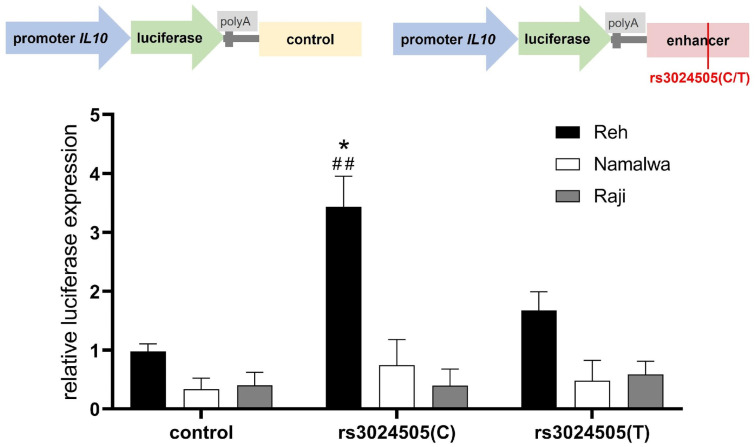
The presence of a risk rs3024505(T) allele abrogates interleukin 10 (IL10) enhancer activity in stimulated Reh cells. Up: Enhancer region containing common (C) or minor (T) alleles of rs3024505 or irrelevant non-enhancer control fragment was cloned downstream of the luciferase gene placed under the *IL10* promoter in the pGL3-basic vector. Down: Relative luciferase activity in Reh, Namalwa, and Raji cells transfected with constructs containing *IL10* promoter and enhancer with alternative rs3024505 alleles or control sequence. All data were normalized to Renilla luciferase internal reference. Data from three independent experiments with mean values ± SEM, * *p* < 0.05—significant difference between SNP alleles; ## *p* < 0.01—significant difference between promoter activity in constructs containing enhancer with rs3024505(C) and control sequence, as calculated by two-way ANOVA.

We conducted the functional analysis of the regulatory elements of human *IL10* locus using a double luciferase assay in immortalized pro-B cell line Reh (B cell acute lymphoblastic leukemia, B-ALL) [40], and Namalwa and Raji B cells from Burkitt’s lymphoma, which represent germinal center B cells [41]. The *IL10* promoter (Figure S1), which was also chosen according to the epigenetic marks of promoter regions [42], was cloned into the pGL3-basic vector, and the putative enhancer region of the *IL10* gene was cloned after the luciferase gene polyadenylation signal. Additional variants of the reporter constructs contained the same enhancer region with the minor allele of the rs3024505(T) and a control genomic DNA fragment with no regulatory properties (Figure 1). We then transfected the resulting constructs into B cells and stimulated the cells with a mix of CD40L, CpG, and IL21, which has been shown to be effective in terms of the induction of B lymphocytes with high immunosuppressive capacity, including IL10 secretion [43].

As a result, the *IL10* promoter appeared to be more active in the Reh cell line than in the Namalwa and Raji cells (Figure 1). Similarly, the reporter vector with the putative enhancer containing the common rs3024505(C) allele demonstrated a 3.5-fold increase in luciferase expression compared to the vector containing an irrelevant sequence in only Reh cells. The conversion of the rs3024505(C) variant to the minor T allele abrogated *IL10* enhancer activity.

### 2.2. The Risk rs3024505(T) Allele Disrupts Functional STAT3 Binding Site

**Figure 2:**
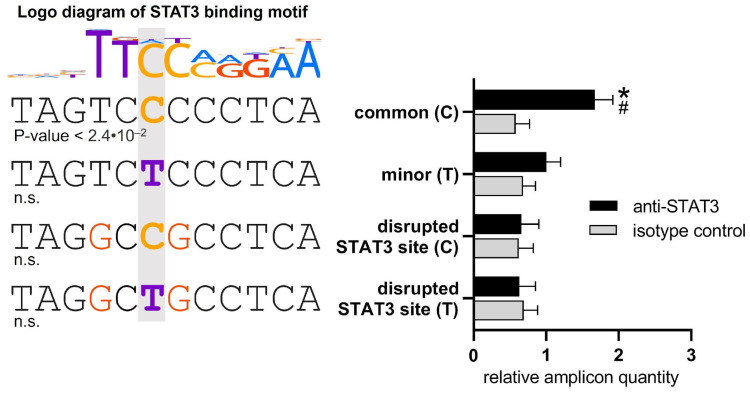
The common allele rs3024505(C) of enhancer creates a STAT3 binding site in stimulated Reh cells. (**Left**): The STAT3 motif logo from the HOCOMOCO v11 database aligned with the corresponding segments of the enhancer sequence with alternative rs3024505(C/T) alleles (highlighted in grey area) and mutated variants with a disrupted STAT3 binding site (nucleotide substitutions are in red). The motif *p*-values are indicated below the sequences, n.s.—non-significant. (**Right**): Pull-down assay with anti-STAT3 antibodies and nuclear extract from stimulated Reh cells. The amplicon amount was quantified by real-time qPCR, normalized to the value obtained with the negative control DNA fragment, and further normalized to the relative amplicon quantity of rs3024505(T) with anti-STAT3. The presented data represent three independent experiments with mean values ± SEM. * *p* < 0.05—compared to other amplicons precipitated by anti-STAT3, # *p* < 0.05—compared to isotype control, as calculated by two-way ANOVA.

Changes in the sequence of the transcription factor (TF) binding site within the region overlapping the SNP can alter the activity of regulatory elements [20]. According to the ADASTRA database [44], the rs3024505(C) creates an allele-specific STAT3 binding site that is disrupted in the presence of the risk T allele. To assess this allele-specific binding, we performed a DNA pull-down assay using nuclear extracts from stimulated Reh cells that were incubated with DNA probes containing an enhancer sequence overlapping rs3024505, followed by immunoprecipitation with antibodies against STAT3. The real-time qPCR quantification of the enriched amplicons revealed that anti-STAT3 antibodies effectively precipitated the amplicons with the common rs3024505(C) allele, which has been predicted to create a STAT3 binding site (Figure 2, left). In contrast, DNA probes carrying the risk rs3024505(T) variant showed no significant precipitation. Similarly, both variants containing point mutations designed to disrupt STAT3 binding exhibited negligible precipitation (Figure 2, right).

### 2.3. The IL10 Enhancer Exhibits Functional Activity upon STAT3 Binding

**Figure 3:**
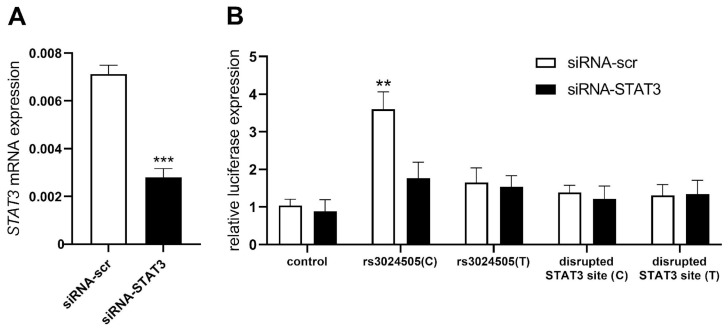
STAT3 knockdown removes the rs3024505 allele-dependent difference in the activity of the *IL10* enhancer in stimulated Reh cells. (**A**) Relative expression levels of STAT3 mRNA in stimulated Reh cells transfected with anti-STAT3 siRNA and control scrambled siRNA (siRNA-scr) according to qRT-PCR data. The data were normalized to β-actin and further normalized to the expression levels mRNA with control scrambled siRNA. Data represent at least three independent experiments with mean values ± SEM. *** *p* < 0.001, as calculated by unpaired Student’s *t*-test. (**B**) Relative luciferase activity in cells transfected with constructs containing the *IL10* promoter and enhancer with alternative rs3024505 alleles or control non-enhancer sequences, and also transfected with anti-STAT3 siRNA and control scrambled siRNA (siRNA-scr). All data were normalized to Renilla luciferase internal reference. Data represent at least three independent experiments with mean values ± SEM. ** *p* < 0.01 compared to all the others, as calculated by two-way ANOVA.

To further validate the hypothesis that the regulatory effect of rs3024505 on *IL10* expression is mediated by altered STAT3 binding, we performed the siRNA-mediated knockdown of *STAT3* in stimulated Reh cells. *STAT3* expression was suppressed by approximately 60% (Figure 3A), resulting in the equalization of enhancer activity between variants carrying alternative rs3024505 alleles in stimulated Reh cells, as measured by reporter expression (Figure 3B). Furthermore, the common rs3024505(C) allele did not affect enhancer activity when point mutations disrupted the STAT3 binding site (Figure 3B). This pattern of reporter activity mirrored the in vitro binding affinity of STAT3 (Figure 2), suggesting that the enhancer activity of the *IL10* gene associated with the common rs3024505(C) allele in the B cell model can be attributed to the presence of a functional STAT3 binding site.

## 3. Discussion

The role of non-coding polymorphisms in the regulation of gene expression and disease development is becoming increasingly recognized. This study investigated the impact of the polymorphism rs3024505, an eQTL for the *IL10* gene implicated in autoimmune disorders. Prior research has demonstrated a genetic association between the minor rs3024505(T) allele and several autoimmune diseases, including systemic lupus erythematosus, Sjögren’s syndrome, Crohn’s disease, and ulcerative colitis. Our reporter assay data revealed that rs3024505 resides within an enhancer of the *IL10* gene. This enhancer exhibited activity in stimulated pro-B cell line Reh, with the rs3024505(T) variant exhibiting a significant reduction in enhancer/promoter activity. Given that genetic variants can impact regulatory element activity by modifying transcription factor binding, we conducted an in silico prediction analysis using the ADASTRA database to identify potential molecular factors. Our analysis identified STAT3 as a promising candidate, and subsequent validation in stimulated Reh cells confirmed its functional role in regulating IL10 expression. This is consistent with allele-specific binding of STAT3 to the rs3024505 region, as demonstrated by DNA affinity precipitation assays with nuclear extracts from polarized Th17 cells [45]. Additionally, a recent murine study reported the downregulation of *IL10* transcription upon the deletion of STAT3 in CD19^+^ B cells [46].

Taken together, our results suggest that the *IL10* enhancer activity associated with the common rs3024505(C) allele in B cells may be attributed to the presence of a functional STAT3 binding site. Intriguingly, we observed this effect only in the stimulated pro-B cell line Reh derived from acute lymphoblastic leukemia (ALL), but not in Namalwa or Raji cells originating from Burkitt’s lymphoma. We hypothesize that this difference may be attributed to the presence of the TEL-AML1 translocation in Reh cells, a characteristic feature of ALL that leads to the aberrant activation of STAT3 and subsequent *IL10* expression [47,48]. Furthermore, this distinct IL10 regulation may be attributed to its pivotal role in pro-B cell survival and development [49]. Another potential explanation is the possible differentiation of activated pro-B cell Reh into monocyte-like cells [50,51]. These cells have the capacity to polarize into alternatively activated macrophages (M2), which actively secrete anti-inflammatory cytokines under the autocrine influence of IL10, probably in a STAT3-dependent manner [52,53]. We also suggest that the regulatory area surrounding rs3024505 could be involved in the transcriptional regulation of nearby genes belonging to the IL10 family of cytokines (*IL19*, *IL20*, *IL24*, etc.), given the existence of published works supporting this hypothesis [33,54,55].

**Figure 4:**
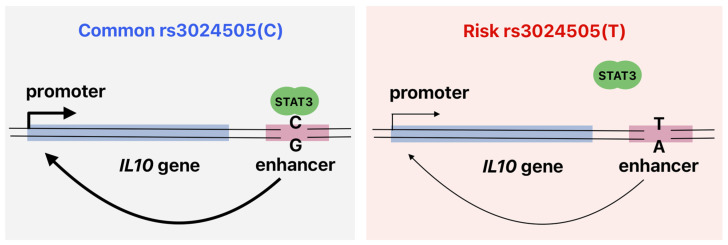
Common rs3024505(C) variant creates a STAT3 binding site, promoting *IL10* enhancer activity in human B cells. The absence of STAT3 binding to the risk rs3024505(T) allele may lead to reduced IL10 production by B cells, potentially disrupting immune homeostasis and increasing the susceptibility to autoimmune diseases.

The suppressive capacity of IL10^+^ regulatory B cells has been reported to be diminished or absent in patients with various autoimmune diseases [56,57], suggesting that decreased IL10 expression may be a critical factor in the pathogenesis of these disorders. The heterogeneous population of regulatory B cells comprises a number of subsets of immature lymphocytes [58,59], including pro-B cells, which demonstrated statistically significant results in this study. Previous research has shown that pro-B cells may possess immunosuppressive properties and can protect against autoimmune reactions and graft-versus-host disease [60,61]. The association of rs3024505(T) with several autoimmune diseases likely arises as a consequence of its contribution to STAT3-mediated *IL10* downregulation (Figure 4). It is noteworthy that the major rs3024505(C) allele is, on the contrary, considered a risk factor for T1D, as indicated by the meta-analysis of polymorphism associations [35,36]. This phenomenon may be attributed to the pathogenic role of IL10 at the initial stages of the disease, with its local release potentially accelerating the development of T1D [15].

Further functional analysis of rs3024505 in gene-edited cell models would be valuable for a more comprehensive understanding of the observed regulatory mechanism.

## 4. Materials and Methods

### 4.1. Cell Cultures

Reh, Namalwa, and Raji cells line were cultivated in RPMI 1640 medium (PanEco, Moscow, Russia) supplemented with 10% FBS (Corning, New York, NY, USA), 2 mM L-glutamine, 1mM sodium pyruvate, 100 U/mL penicillin, 100 μg/mL streptomycin (all PanEco, Moscow, Russia), non-essential amino acids, and 10 mM HEPES (all Gibco, Kwartsweg, The Netherlands). The day before electroporation with test plasmids, cells were treated with a mixture of recombinant CD40L (1 μg/mL, BioLegend, San Diego, CA, USA), IL21 (25 ng/mL, Miltenyi Biotec, Bergisch Gladbach, Germany), and CpG (2 μM, ODN2006, 5′-tcgtcgttttgtcgttttgtcgtt-3′).

### 4.2. Reporter Plasmids

To analyze the activity of *IL10* regulatory elements, we amplified the putative *IL10* promoter region (chr1:206772325-206775043; GRCh38/hg38), putative enhancer elements including rs3024505 (chr1:206766108-206767105; GRCh38/hg38), and a similarly sized irrelevant control sequence (a previously published intronic region of the *STAT3* gene with no epigenetic features of the regulatory area; chr17:42356476-42357555; GRCh38/hg38 [62]) by PCR using human genomic DNA (Promega, Madison, WI, USA) as a template and specific primers containing restriction sites (Table S3). The promoter was cloned into a pGL3-basic luciferase reporter construct (Promega, Madison, WI, USA) using BglII and PagI/NcoI restriction sites, and the enhancer or control sequence was cloned using BamHI and SalI restriction sites downstream of the luciferase coding sequence and polyadenylation signal. To analyze the influence of rs3024505(C/T), site-specific nucleotide changes in enhancer regions were introduced by two-stage PCR using internal overlapping primers (Table S3). Plasmid DNA was extracted and purified with the NucleoBond Xtra Midi Kit (Macherey-Nagel, Düren, Germany) and verified by Sanger sequencing (EIMB RAS “Genome” center, Moscow, Russia).

### 4.3. Cell Transfection and Luciferase Reporter Assay

For the luciferase assay, 2.5 mln cells were transfected with 5 μg of test plasmids combined with 0.5 μg of pRL-CMV Renilla luciferase control vector from the Dual-Luciferase Reporter Assay System (Promega, Madison, WI, USA). Cells were electroporated using the Neon Transfection System (Invitrogen, Carlsbad, CA, USA) with appropriate parameters: three 10 ms 1400 V pulses for Reh cells, one 30 ms 1300 V pulse for Raji cells, and two 20 ms 1350 V pulses for Namalwa. Luciferase activity was measured 24 h post-transfection using a 20/20n Luminometer (TurnerBioSystems, Sunnyvale, CA, USA).

### 4.4. siRNA-Mediated STAT3 Knockdown

We used previously published short interfering RNA (siRNA) against the *STAT3* gene [63], and appropriate sequences of the control scrambled RNA (siRNA-scr) were obtained by the Invivogen siRNA Wizard v3.1 tool (Table S3). Single-stranded RNAs were commercially synthesized and annealed as previously described [64]. For *STAT3* genetic knockdown, 3 mln cells were transfected by electroporation (as described above) with a 500 pmol mix of STAT3-specific siRNAs or siRNA-scr duplexes. Cells were cultured for 48 h and then were transfected with 5 μg of test vectors, 0.5 μg of pRL-CMV control, and 300 pmol more of siRNA or siRNA-scr. The luciferase reporter assay was conducted after cell cultivation for 24 h. 

### 4.5. DNA Pull-Down Assay

We performed a pull-down assay based on the method described earlier [65]. The “pull-down” primers (Table S3) were designed to produce 154 bp amplicons, corresponding to the sequences with minor and common rs3024505 variants; plasmids containing enhancer elements with different nucleotide variants were used as templates for the PCR reaction. Control amplicon was produced by PCR amplification with the “pull-down-control” primers pair (Table S3), including a 161 bp with no predicted STAT3 binding sites. All PCR products were verified by Sanger sequencing. Nuclear extracts were prepared from stimulated Reh cells using NE-PER Nuclear and Cytoplasmatic Extraction Reagents (Thermo Fisher Scientific, Rockford, IL, USA). Amplicons were incubated with 1 μg STAT3 Mouse mAb IgG (GB12176-100, ServiceBio, Wuhan, China) or 1 μg Rabbit mAb IgG XP Isotype Control (DA1E, Cell Signaling Technology, Inc., Danvers, MA, USA) as an isotypic control. The immunoprecipitation of DNA–protein complexes was performed using Protein A Mag Sepharose (GE Healthcare, Chalfont St Giles Buckinghamshire, UK). Eluted DNA was quantified by qPCR in real-time using “pull-down” primers.

### 4.6. RNA Extraction, cDNA Synthesis and qPCR in Real-Time

Total RNA was isolated using the TRIzol reagent (Invitrogen, Carlsbad, CA, USA) as described in the manufacturer’s manual. The reverse transcription of total RNA was carried out using the MMLV RT Kit (Evrogen, Moscow, Russia) and Oligo(dT)_15_ as primers. Real-time PCR analysis was performed using the CFX96 Touch Real-Time PCR Detection System (Bio-Rad, Hercules, CA, USA) and qPCRmix-HS SYBR (Evrogen, Moscow, Russia). β-Actin (*ACTB*) was used as a reference gene for *STAT3* expression. The pull-down amplicon amount was normalized to the value obtained with the negative control DNA fragment. Sequences of oligonucleotide primers are presented in Table S3.

### 4.7. Statistical Analysis

We performed statistical analysis using GraphPad Prism 9 software. Statistical significance was estimated using unpaired Student’s *t*-test or two-way ANOVA. *p*-values < 0.05 were considered significant. Data for each sample represent the result of at least three independent experiments. Real-time qPCR and luciferase assays were additionally performed in two technical replicates. All data are presented as mean ± SEM.

## Data Availability

The raw data supporting the results presented in this study are available upon request from the corresponding author.

## Supplementary Materials

The following supporting information can be downloaded at https://www.mdpi.com/article/10.3390/ijms251810196/s1.
