# Supplementary material for: Autoimmunity-Associated SNP rs3024505 Disrupts STAT3 Binding in B Cells, Leading to IL10 Dysregulation
Source: Int J Mol Sci. 2024 Sep 23;25(18):10196. doi: 10.3390/ijms251810196 (PMC11432243; doi:10.3390/ijms251810196)
Supplement: Supplementary file 1 [file ijms-25-10196-s001.zip › ijms-3161155-supplementary.pdf]

# Supplementary Materials

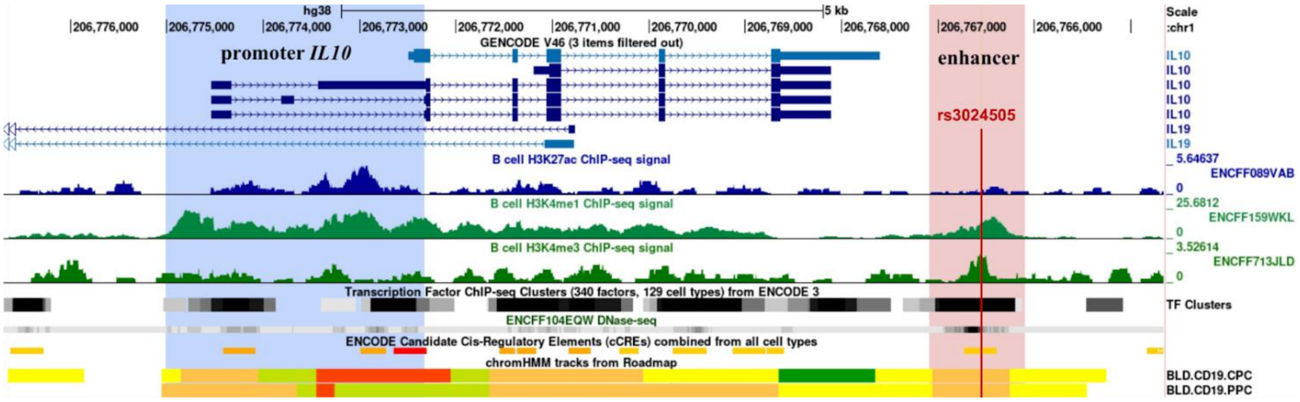


**Figure S1. Schematic representation of the regulatory elements of *IL10* gene and SNP rs3024505 based on the UCSC Genome Browser visualization (GRCh38/hg38, reverse).** The light blue area highlights the putative *IL10* promoter region, the light red – putative enhancer, and the vertical red line shows the location of the rs3024505. Histograms indicate the location of histone modifications in B cells typical of regulatory elements (H3K4 mono/tri-methylation, H3K27 acetylation; Roadmap epigenomic data). Chromatin state segmentation in different subsets of B cells represents ChromHMM model (Roadmap): red and orange denotes a state with a promoter-like signature, yellow denote states with an enhancer-like signature, green indicates a signature for a transcribed region. Rectangles mark regulatory elements positions according to ENCODE, DNase I hypersensitivity clusters (DNase I) in B cells (Roadmap), and transcription factor ChIP-seq (TF clusters; ENCODE).

**Table S1.** rs3024505 is associated with autoimmune pathologies.

| Pathologies associated with the minor rs3024505(T) allele | Populations (**odds ratio, OR**), minor allele frequencies in cases and healthy controls | References |
|---|---|---|
| Systemic lupus erythematosus | European American (**1.30**):<br>0.18 – case<br>0.15 – control<br><br>United States (**1.14**):<br>0.17 – case<br>0.15 – control<br><br>Sweden (**1.21**):<br>0.18 – case<br>0.15 – control | [s1,2] |
| Crohn's disease | New Zealand population (**1.37**):<br>0.21 – case<br>0.16 – control<br><br>Danish population (**1.34**):<br>0.23 – case<br>0.18 – control<br><br>Serbian population (**1.82**):<br>0.22 – case<br>0.14 – control | [s3-5] |
| Ulcerative colitis | Australian population (**1.22**):<br>0.18 – case<br>0.15 – control<br><br>Danish population (**1.37**):<br>0.23 – case<br>0.18 – control | [s4,6] |
| Sjögren's syndrome | Italian population (**1.52**):<br>0.17 – case<br>0.12 – control | [s7] |

**Table S2.** rs3024505 minor allele frequencies for different populations (NCBI dbSNP).

| Minor allele frequencies | Global | American | African | East Asian | European | South Asian |
|---|---|---|---|---|---|---|
| 1000Genomes_30x | 0.086 | 0.081 | 0.034 | 0.03 | 0.164 | 0.141 |
| gnomAD | 0.113 | 0.083 | 0.047 | 0.026 | 0.158 | no data |
| ALFA | 0.144 | no data | 0.045 | 0.027 | 0.155 | 0.122 |

**Table S3.** Oligonucleotides utilized in the study.

| Oligonucleotide name | Sequence (5′→3′) |
|---|---|
| **Primers for amplification of IL10 promoter, enhancer and site-directed mutagenesis** | |
| IL10_prom_BglII_F | TTTAGATCTGTCAGATCCTAACCTCAACCCT |
| IL10_prom_PagI_R | TAATCATGATAGGCAGGTTGCCTG |
| IL10_enh_BamHI_F | TGTGGATCCCGGCTAATTTTTTGCATTTT |
| IL10_enh_SalI_R | TAAGTCGACAGGTAGGTAAGAGACAAATGG |
| Enh_control_BamHI_F | AGGATCCGGATTACAGGTGTATTTCACCAT |
| Enh_control_SalI_R | TATGTCGACGTTGATGTAATTCCTTTAAATCTAT |
| rs3024505(T)_F | TAAACACTAGTCTCCCTCACG |
| rs3024505(T)_R | CGTGAGGGAGACTAGTGTTTA |
| mutSTAT3-rs3024505(T)_F | TAAACACTAGGCTGCCTCACG |
| mutSTAT3-rs3024505(T)_R | CGTGAGGCAGCCTAGTGTTTA |
| mutSTAT3-rs3024505(C)_F | TAAACACTAGGCCGCCTCACG |
| mutSTAT3-rs3024505(C)_R | CGTGAGGCGGCCTAGTGTTTA |

| Primers for amplification of DNA probes for pull-down assay | |
|---|---|
| pull-down-rs3024505_F | AGGGGCACGGTGGCTGGGGA |
| pull-down-rs3024505_R | ACTGACCACAGCCTGTCCGC |
| pull-down-control_F | AACTCCTGGCCTCAAGTGATC |
| pull-down-control_R | TAGTGCCAGAAGCCCTGTTAG |
| **siRNAs for STAT3 knockdown and primers for qPCR in real-time** | |
| siRNA1-STAT3_sense | AACAUCUGCCUAGAUCGGCUAdTdT |
| siRNA1-STAT3_antisense | UAGCCGAUCUAGGCAGAUGUUdTdT |
| siRNA2-STAT3_sense | GGUACAUCAUGGGCUUUAUdTdT |
| siRNA2-STAT3_antisense | AUAAAGCCCAUGAUGUACCdTdT |
| scrambled_RNA_sense | GACCGCAUUCUACGAGACUAUdTdT |
| scrambled_RNA_antisense | AUAGUCUCGUAGAAUGCGGUCdTdT |
| rtpcr_STAT3_F | CTTTGAGACCGAGGTGTATCACC |
| rtpcr_STAT3_R | GGTCAGCATGTTGTACCACAGG |

**Supplementary references**


[s1]. Gateva, V.; Sandling, J.K.; Hom, G.; Taylor, K.E.; Chung, S.A.; Sun, X.; Ortmann, W.; Kosoy, R.; Ferreira, R.C.; Nordmark, G.; et al. A Large-Scale Replication Study Identifies TNIP1, PRDM1, JAZF1, UHRF1BP1 and IL10 as Risk Loci for Systemic Lupus Erythematosus. *Nat. Genet.* **2009**, *41*, 1228–1233, doi:10.1038/ng.468.

[s2]. Sakurai, D.; Zhao, J.; Deng, Y.; Kelly, J.A.; Brown, E.E.; Harley, J.B.; Bae, S.-C.; Alarcón-Riquelme, M.E.; BIOLUPUS and GENLES networks; Edberg, J.C.; et al. Preferential Binding to Elk-1 by SLE-Associated IL10 Risk Allele Upregulates IL10 Expression. *PLoS Genet.* **2013**, *9*, e1003870, doi:10.1371/journal.pgen.1003870.

[s3]. Wang, A.H.; Lam, W.-J.; Han, D.-Y.; Ding, Y.; Hu, R.; Fraser, A.G.; Ferguson, L.R.; Morgan, A.R. The Effect of IL-10 Genetic Variation and Interleukin 10 Serum Levels on Crohn's Disease Susceptibility in a New Zealand Population. Hum. Immunol. 2011, 72, 431–435, doi:10.1016/j.humimm.2011.02.014.

[s4]. Andersen, V.; Ernst, A.; Christensen, J.; Østergaard, M.; Jacobsen, B.A.; Tjønneland, A.; Krarup, H.B.; Vogel, U. The Polymorphism rs3024505 Proximal to IL-10 Is Associated with Risk of Ulcerative Colitis and Crohns Disease in a Danish Case-Control Study. BMC Med. Genet. 2010, 11, 82, doi:10.1186/1471-2350-11-82.

[s5]. Mijac, D.; Petrovic, I.V.; Djuranovic, S.; Perovic, V.; Bojic, D.; Culafic, D.; Popovic, D.; Krstic, M.; Jankovic, G.; Djoric, M.; et al. The Polymorphism rs3024505 (C/T) Downstream of the IL10 Gene Is Associated with Crohn's Disease in Serbian Patients with Inflammatory Bowel Disease. Tohoku J. Exp. Med. 2016, 240, 15–24, doi:10.1620/tjem.240.15.

[s6]. Doecke, J.D.; Simms, L.A.; Zhao, Z.Z.; Huang, N.; Hanigan, K.; Krishnaprasad, K.; Roberts, R.L.; Andrews, J.M.; Mahy, G.; Bampton, P.; et al. Genetic Susceptibility in IBD: Overlap between Ulcerative Colitis and Crohn's Disease. *Inflamm. Bowel Dis.* **2013**, *19*, 240–245, doi:10.1097/MIB.0b013e3182810041.

[s7]. Colafrancesco, S.; Ciccacci, C.; Priori, R.; Latini, A.; Picarelli, G.; Arienzo, F.; Novelli, G.; Valesini, G.; Perricone, C.; Borgiani, P. STAT4, TRAF3IP2, IL10, and HCP5 Polymorphisms in Sjögren's Syndrome: Association with Disease Susceptibility and Clinical Aspects. *J Immunol Res* **2019**, *2019*, 7682827, doi:10.1155/2019/7682827.
